# Hypereosinophilic Syndrome with Pulmonary Involvement in Ulcerative Colitis

**DOI:** 10.1097/PG9.0000000000000320

**Published:** 2023-06-09

**Authors:** Anam Bashir, Jose M. Cabrera, Mariko Suchi, Barry J. Pelz

**Affiliations:** From the * Division of Pediatric Gastroenterology, Hepatology and Nutrition, Department of Pediatrics, Medical College of Wisconsin, Milwaukee, WI; † Associate Professor, Division of Pediatric Gastroenterology, Hepatology and Nutrition, Department of Pediatrics, Medical College of Wisconsin, Milwaukee, WI; ‡ Associate Professor, Department of Pathology and Laboratory Medicine, Medical College of Wisconsin, Milwaukee, Wisconsin, WI; § Assistant Professor, Division of Allergy and Immunology, Department of Pediatrics, Medical College of Wisconsin, Milwaukee, WI

**Keywords:** ulcerative colitis, hypereosinophilic syndrome, eosinophilia

## Abstract

Reactive eosinophilia is associated with inflammatory bowel disease and is more common in patients with ulcerative colitis (UC) compared with Crohn’s disease. The prevalence rate of peripheral blood eosinophilia in patients with inflammatory bowel disease has been described to be as high as 30%–40% of patients in a pediatric study. The coexistence of hypereosinophilic syndrome (HES) and UC is uncommon. We present a 15-year-old boy with UC associated with HES who presented with chest pain and shortness of breath. Laboratory evaluation showed marked eosinophilia. Alternative causes of eosinophilia including eosinophilic leukemia, infections, or drug-induced eosinophilic pneumonia were ruled out. The patient was ultimately diagnosed with HES responsive to mepolizumab.

## INTRODUCTION

Patients with inflammatory bowel disease (IBD), especially ulcerative colitis (UC), frequently have reactive eosinophilia, which has been linked to a more severe disease phenotype. The prevalence rate of peripheral blood eosinophilia in patients with IBD has been described to be as high as 30%–40% of patients in a pediatric study (1). The coexistence of hypereosinophilic syndrome (HES) and UC has rarely been described before (2–5). We present a 15-year-old boy with UC associated with HES who presented with chest pain, shortness of breath, and marked eosinophilia.

## CASE REPORT

**FIGURE 1.**
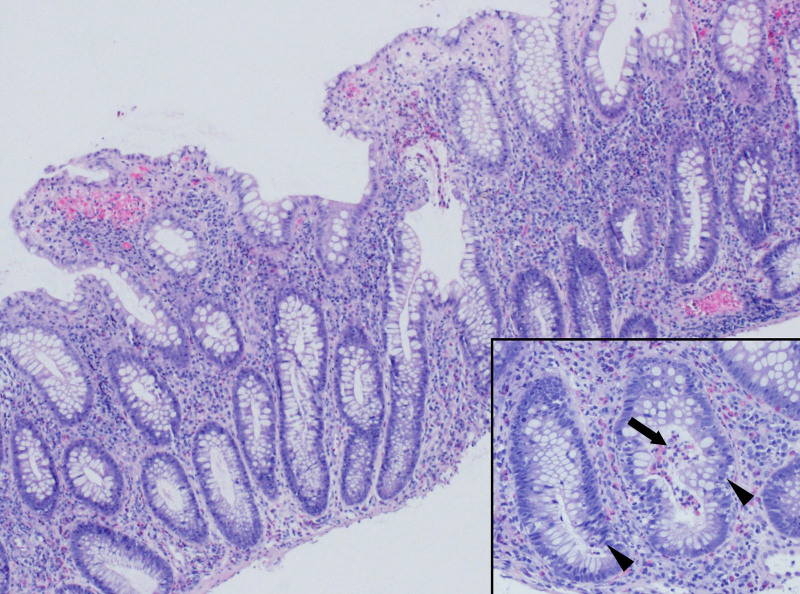
Microphotographs of colonic biopsy. The colonic mucosa demonstrates elongated and bifurcated crypts within markedly cellular lamina propria containing increased lymphocytes, plasma cells, eosinophils, and neutrophils. Foci of cryptitis (arrowheads) and a crypt abscess (arrow) are shown in the inset. Hematoxylin and eosin, original magnification ×40, inset ×100.

**FIGURE 2.**
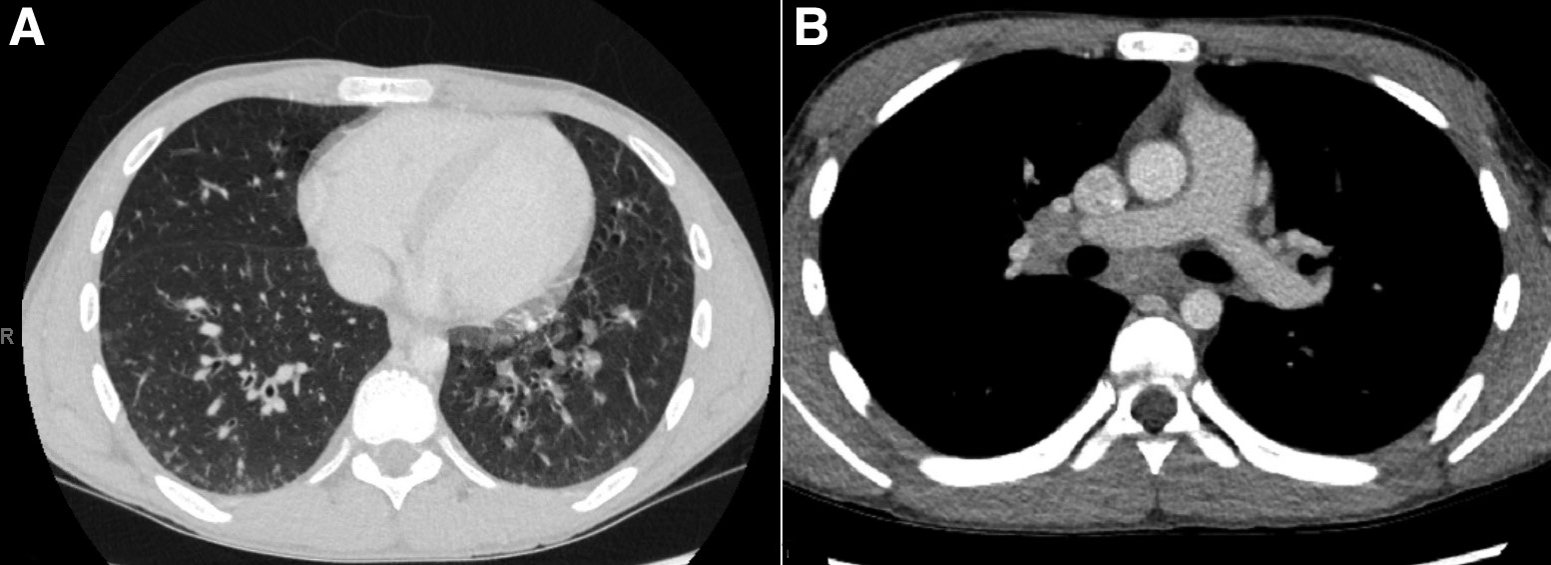
Chest computed tomography at presentation (A) Bibasilar consolidations and ground glass opacities are seen, mild bibasilar predominant bronchial wall thickening (B) Enlarged right hilar and mediastinal lymph nodes.

**FIGURE 3.**
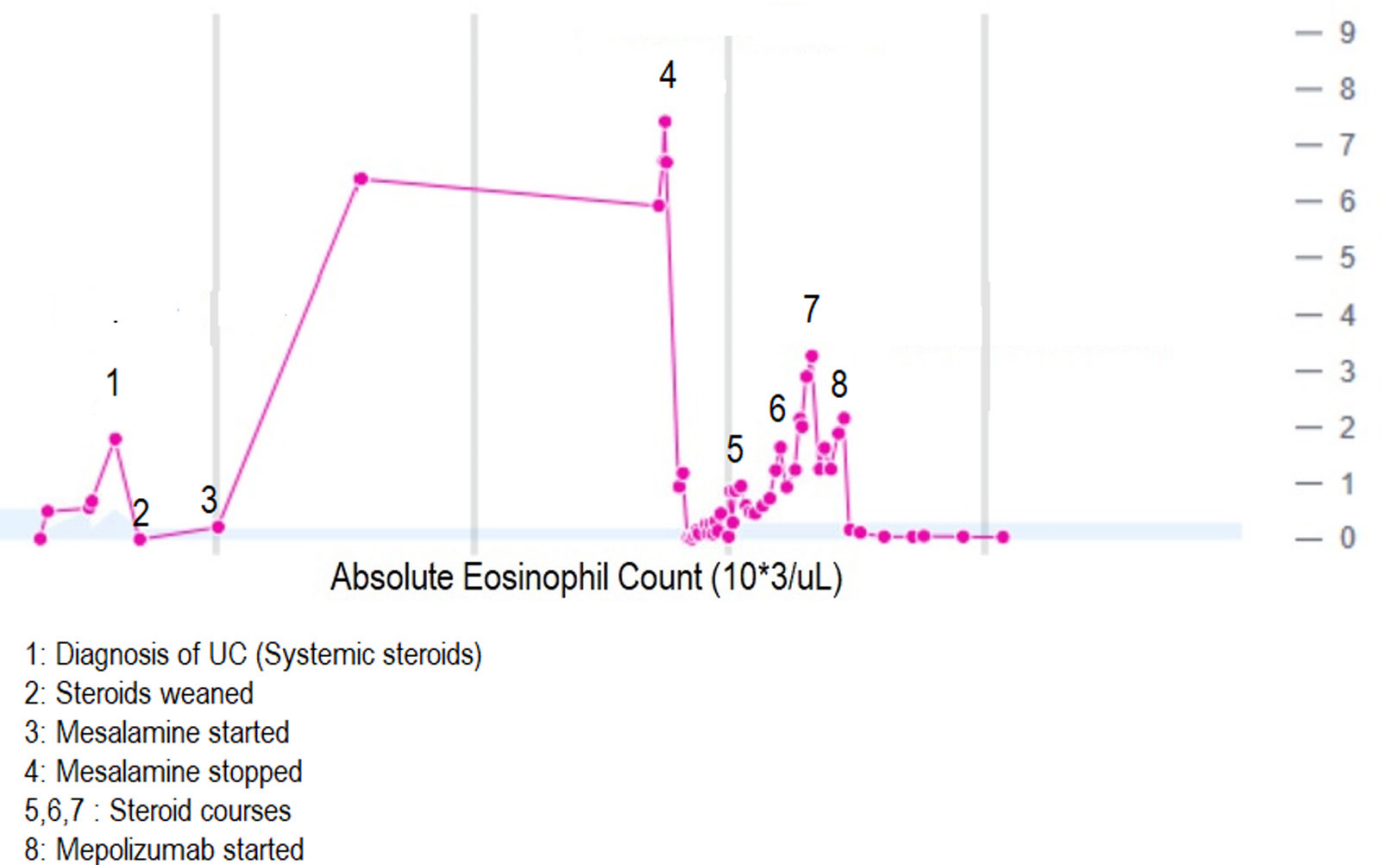
Absolute eosinophil count progression at diagnosis of ulcerative colitis (UC), discontinuation of mesalamine, and after treatment with mepolizumab treatment.

A 15-year-old male with a history of asthma and allergic rhinitis presented with bloody diarrhea, abdominal pain, and weight loss. Colonoscopy with biopsy revealed diffuse active chronic colitis (Fig. 1) from the cecum to the rectum without terminal ileum involvement. Initial treatment with mesalamine resulted in good control of symptoms. After 2 years of treatment, he was admitted to our hospital for chest pain and progressive shortness of breath. He had presented to urgent care 2 weeks prior and received ipratropium bromide/albuterol nebulization and outpatient oral corticosteroids for presumed “asthma exacerbation”. Coronavirus disease and Influenza A and B Nucleic Acid Amplification Tests were negative. He reported similar episodes of cough, congestion, and dyspnea episodically over the past year with each episode being attributed to frequent asthma exacerbations. Home medications included mesalamine 4800 mg (92 mg/kg/day) once daily fluticasone propionate/salmeterol 500/50 mcg one puff twice daily, albuterol 90 mcg/puff inhaler prn, and montelukast 10 mg nightly. Physical examination revealed age-appropriate vital signs including normal oxygen saturation in room air. Chest examination revealed expiratory wheezing in all lung fields. Complete blood cell count showed elevated white blood cell count of 19.2 × 10^3^/μl (49.5% neutrophils, 9.3 % lymphocytes, 1.6% monocytes, 38.7% eosinophils, and 0.3% basophils). The absolute eosinophilic count was 6.69 × 10^3^/μl. The erythrocyte sedimentation rate was mildly elevated at 26 mm/h (0–10 mm/h). Due to the eosinophilia, other laboratory tests including troponin I level, angiotensin-converting enzyme level, and tryptase level were obtained and were normal. Antiproteinase 3 and antimyeloperoxidase antibody studies were negative. Immunoglobulin E (IgE) was elevated (1867 kU/L); other immunoglobulin levels were normal. The antinuclear antibody test was negative, and rheumatoid factor was not obtained. Peripheral blood smear showed normochromic normocytic red blood cells, normal platelets, and white blood cells increased in number with eosinophilia (36%). Chest X-ray was normal. Stool ova and parasites were negative. Flow cytometry of the bone marrow aspirate showed no clonal expansion of cells fluorescence in-situ hybridization investigating PDGFRA-FIP1L1 rearrangements and CHIC2 deletions was negative. Electrocardiogram was normal. Chest computed tomography (CT) showed bibasilar predominant scattered tiny consolidations and ground glass opacities, mild bibasilar predominant bronchial wall thickening, and a few mildly prominent right hilar and mediastinal lymph nodes (Fig. 2). With peripheral eosinophilia and chest CT findings with an otherwise nondiagnostic evaluation, his symptoms were attributed to mesalamine causing eosinophilic pneumonia. Mesalamine was discontinued and he was started on vedolizumab for UC. He was also started on 60 mg prednisone daily, with improvement in symptoms. Repeat complete blood cell count of the outpatient showed normal white blood cell count with decreased eosinophilic count (0.1%). The absolute eosinophil count was 0.01 × 10^3^/μl. As corticosteroids were weaned over the next 10 weeks to 15 mg daily, he had a recurrence of shortness of breath and cough without gastrointestinal (GI) symptoms, and peripheral blood eosinophilia with an absolute eosinophilia count of 1646 × 10^3^/μl (16.8%). He was restarted on a 5-day course of corticosteroids 40 mg with improvement. After another attempt to wean corticosteroids, eosinophilia increased again up to 3267 × 10^3^/μl (33%) with the return of symptoms. With continued eosinophilia despite stopping mesalamine (more than 3 months out from discontinuation), he was referred to oncology for further evaluation. Bone marrow biopsy showed hypercellularity with increased eosinophils (22%) and their precursors without increased blast cells. With hypereosinophilia (absolute eosinophilic count >1.5 × 10^9^/L in peripheral blood and >20% of eosinophils on bone marrow aspirate) and organ damage as evidenced by pulmonary symptoms, he was ultimately diagnosed with HES. With continued eosinophilia, he was started on mepolizumab 300 mg subcutaneously every 4 weeks, while continuing vedolizumab for UC. Eosinophilia resolved after 2 injections of mepolizumab (Fig. 3). He has developed no recurrent cough, shortness of breath, or corticosteroid requirement for his symptoms since initiating mepolizumab 6 months earlier.

## DISCUSSION

Our report of an adolescent with UC with significant eosinophilia demonstrates the importance of understanding the consequences of eosinophilia in these patients. Eosinophils differentiate in the bone marrow under the influence of interleukin 3 (IL-3), interleukin 5 (IL-5), and granulocyte colony-stimulating factors and primarily reside in the stomach and intestine. Eosinophils are known to be in excess in IBD and get recruited more to the GI tract because of increased expression of eotaxin and IL-5, which are overexpressed in IBD (6,7). Peripheral eosinophilia in UC has been associated with more severe disease and a higher likelihood of other conditions such as primary sclerosing cholangitis and overlap autoimmune hepatitis (6). Because of its association with eosinophilia, UC has been postulated to be an allergic disorder.

HESs are a group of disorders characterized by peripheral blood eosinophilia of 1.5 × 10^3^/μl or greater with evidence of end-organ damage attributable to eosinophilia. Primary HES is caused by an underlying neoplasm of stem cells, eosinophils, or myeloid cells. Secondary HES is caused by the overproduction of eosinophilopoietic cytokines which can be provoked by parasites, allergies, infections, solid organ tumors, or underlying T-cell lymphoma. Idiopathic HES is the subtype where the etiology remains unknown despite thorough evaluation. Common target organs include the skin, lungs, and GI tract. The estimated prevalence is 0.36–6.3 per 100,000 (8). The goal of treatment is to reduce eosinophilia to prevent further organ damage. Corticosteroids are the first line of treatment. For patients refractory to corticosteroids, alternative therapies include azathioprine, cyclosporine, methotrexate, rituximab, hydroxyurea, imatinib, and thalidomide. These are rapidly being replaced in favor of antieosinophilic biologic agents including mepolizumab.

It is possible that our patient’s bowel findings are due to HES even though histologic findings are typical of UC. It is difficult to distinguish between single-organ overlap HES (limited to one organ like the GI tract), primary eosinophilic gastrointestinal disease (EGID), and reactive eosinophilia presenting as GI eosinophilia. EGID has florid eosinophilic infiltration of the GI tract, unlike our case. Other organ involvement besides the GI tract is uncommon in EGID (9).

Mesalamine-related lung injury can manifest as interstitial and eosinophilic pneumonitis, bronchiolitis obliterans, and eosinophilic pleural effusion. Symptoms and radiologic findings usually resolve within days to weeks from mesalamine discontinuation (10,11). Our patient continued to demonstrate symptoms, including cough and shortness of breath despite discontinuation of mesalamine for 4 months. He required frequent corticosteroid courses which led us to consider additional etiologies for his pulmonology symptoms. Pulmonary involvement as a complication of IBD was also a possibility. However, in the absence of extra-intestinal manifestations as well as no active symptoms of UC, we excluded the possibility of pulmonary manifestations of IBD since pulmonary symptoms most often parallel that of GI tract flares, typically occur during exacerbations, and respond to systemic corticosteroids (10–12). Large airway involvement can present when the disease is quiescent, however, this is generally responsive to corticosteroids (11).

HES is potentially life-threatening and can involve multiple organs. Peripheral blood eosinophilia in prior studies has been found to be associated with IBD, mainly with UC (13). Peripheral blood eosinophilia has been demonstrated in 27%, 30%, and 8% of patients at diagnosis, follow-up with histologic activity, and at follow-up with histologic remission (14). Since patients with UC can present with eosinophilia, HES should be part of the differential for eosinophilia in these patients. Severe cases of HES can present with life-threatening complications such as myocardial ischemia and respiratory failure and should be started promptly on eosinophil lowering therapy to prevent disease progression and organ injury (15).
